# Effect of antidepressants on functioning and quality of life outcomes in children and adolescents with major depressive disorder: a systematic review and meta-analysis

**DOI:** 10.1038/s41398-022-01951-9

**Published:** 2022-05-04

**Authors:** Teng Teng, Zhihan Zhang, Bangmin Yin, Tingting Guo, Xiaoya Wang, Jiayi Hu, Xin Ran, Qi Dai, Xinyu Zhou

**Affiliations:** 1 Department of Psychiatry, The First Affiliated Hospital of Chongqing Medical University, Chongqing, China; 2 The First Clinical College of Chongqing Medical University, Chongqing, China; 3 Department of Neurology, People’s Hospital of Deyang City, Deyang, China; 4 The Second Clinical College of Chongqing Medical University, Chongqing, China

**Keywords:** Depression, Clinical pharmacology

## Abstract

Functioning and quality of life (QOL) are typical outcomes assessed in children and adolescents with major depressive disorder (MDD); however, meta-analytical evidence remains scarce. The aim of this meta-analysis was to assess functioning and QOL antidepressant outcomes in this population. Eight electronic databases (PubMed, Cochrane Library, Web of Science, Embase, CINAHL, PsycINFO, LILACS, and ProQuest Dissertation Abstracts) were searched for double-blind randomized controlled trials (RCTs) up to July 31, 2020. RCTs that compared antidepressants with placebo for treating functioning and QOL in children and adolescents with MDD were included. Primary outcomes were mean change scores of functioning and QOL scales from baseline to post-treatment. Subgroup and sensitivity analyses were conducted to examine whether results were affected by moderator variables (e.g., medication type, age, sample size, and treatment duration). From 7284 publications, we included 17 RCTs (all 17 assessed functioning and 4 assessed QOL outcomes) including 2537 participants. Antidepressants showed significant positive effects on functioning (standardized mean difference [SMD] = 0.17, 95% confidence interval [CI] = 0.09–0.25, *p* < 0.0001) but not on QOL (SMD = 0.11, 95% CI = −0.02 to 0.24, *p* = 0.093), with no significant heterogeneity. The subgroup analysis showed that second-generation antidepressants (especially fluoxetine, escitalopram, and nefazodone), but not first-generation antidepressants, led to significant improvements in functioning. Antidepressants (especially second generation) improve functioning but not QOL in children and adolescents with MDD. However, well-designed clinical studies using large samples are needed to confirm these findings.

## Introduction

Major depressive disorder (MDD) is one of the most common and burdensome mental disorders worldwide, with an estimated prevalence of 1.6% in children (aged 8–11 years) and 3.8% in adolescents (aged 12–15 years) [1]. Childhood and adolescence are important periods that are characterized by a high risk of psychiatric disorders. MDD is the second or third leading contributor to disease burden in young people [2]. Compared with adults, children and adolescents with MDD have more serious social and educational functioning impairments as well as poorer quality of life (QOL) [3]. Most clinical trials on pediatric depression have assessed depressive symptoms as the primary outcomes. However, MDD diagnosis and treatment should include the outcomes of functioning and QOL, because both outcomes show substantial impairment in pediatric MDD [4, 5] and contribute to many negative conditions. A recent systematic review found that between 2007 and 2017, 94% of studies on treatment efficacy and effectiveness for adolescent depression tracked depressive symptom changes, 52.2% tracked functioning changes, but only 7.6% tracked QOL changes [6], indicating that a relatively low proportion of clinical trials have focused on functioning and QOL in pediatric MDD.

Functioning refers to the objective or subjective assessment of performance in behavioral domains, such as occupational, social, or family functioning [7]. Specifically, children and adolescents with MDD may have problems dealing with family relationships, behavior at school or work, and interacting with other children [4]. Social functioning impairment may contribute to the high levels of stigma and disability associated with MDD [8]. Furthermore, compared with their peers, children and adolescents with MDD may experience cognitive and social developmental delays owing to impairment in related functioning; even after depression remission, they may experience more difficulty “catching up” to meet developmental milestones [9]. The World Health Organization defines QOL as “individuals’ perception of their position in life in the context of the culture and value systems in which they live, and in relation to their goals, expectations, standards and concerns” [10]. Children and adolescents with lower QOL have lower self-reported satisfaction with physical and psychological well-being and with social support and peers [11]. This early life satisfaction may exert a positive effect on school performance and adult life [12]. Moreover, QOL improvement is an early indicator of differential treatment responses [13] and has a protective effect against recurrence [14].

A previous review reported that early effective treatment for MDD reduces the risk of long-term negative outcomes and has a sustained positive effect on functioning and life satisfaction into adulthood [15]. Antidepressants are widely used to treat MDD in children and adolescents [16]. Antidepressants are recommended for moderate to severe cases of pediatric depression in some pediatric clinical guidelines, such as the UK National Institute for Health and Care Excellence guidelines and the Royal Australian and New Zealand College of Psychiatrists clinical practice guidelines for mood disorders [17, 18]. However, compared with their efficacy for adult patients [19], the efficacy of antidepressant drugs for the treatment of young people with MDD is controversial, as shown in our recent network meta-analysis [20, 21]. Moreover, it is unclear whether children and adolescents with MDD benefit from antidepressants in terms of improvements in functioning and QOL [21].

Thus, we conducted a meta-analysis to synthesize the available evidence and evaluate antidepressant functioning and QOL outcomes in children and adolescents with MDD. These findings will likely have important implications for clinical decisions and policymaking regarding the use of antidepressants in children and adolescents with MDD.

## Methods

### Data sources and searches

For this meta-analysis, we searched eight relevant electronic databases (PubMed, Embase, Cochrane Library, Web of Science, CINAHL, PsycINFO, LILACS, and ProQuest Dissertation Abstracts) from inception to July 2020. Keywords included (depress* or “mood disorder*” or “affective disorder*”) AND (adolesc* or child*) AND (antidepressant* or SSRI or SNRI or NaSSA or TCA). Details of the systematic search strategies and results are provided in Supplementary Table S1. Furthermore, to identify additional eligible randomized controlled trials (RCTs) and reviews, we scanned the reference lists of the relevant studies. We also contacted authors of potentially eligible studies and searched the available trial registration databases. No language restrictions were applied to the searches.

### Study selection

Two independent reviewers (TT, ZZ) selected studies for inclusion; any disagreements were resolved by a third author (XZ). Double-blind RCTs were included if they met all the following criteria: (1) included children and adolescents aged 18 years or younger with a primary diagnosis of MDD based on standardized diagnostic criteria (e.g., Diagnostic and Statistical Manual of Mental Disorders, Third Edition (DSM-III) or Fourth Edition (DSM-IV) and Research Diagnostic Criteria); (2) compared any commonly prescribed antidepressant (used as oral monotherapy) with placebo, including tricyclic antidepressants (TCAs), selective serotonin reuptake inhibitors (SSRIs), and serotonin–norepinephrine reuptake inhibitors (SNRIs), as well as the novel agents mirtazapine and nefazodone, but only if administered within the therapeutic dose range; and (3) measured functioning or QOL outcomes.

RCTs were excluded if (1) they involved patients with treatment-resistant depression or psychotic depression; (2) data overlapped with those reported in another study that was considered for inclusion; (3) they included adult data, and data on children/adolescents could not be extracted separately; and (4) treatment duration was <4 weeks, or the overall sample size was smaller than 10 patients.

### Outcome measures

To measure the functioning and QOL outcomes of antidepressants in patients with MDD, we defined primary outcomes as mean changes in functioning and QOL scale scores from baseline to post-treatment. Included studies used the following functioning scales: Children’s Global Assessment Scale (CGAS) [22], Global Assessment Function (GAF) [23], and Autonomous Functioning Checklist (AFC) [24]. QOL scales included the Pediatric Quality of Life Enjoyment and Satisfaction Questionnaire (PQ-LES-Q) [25], European Quality of Life Scale (EuroQol) [26], and Sickness Impact Profile (SIP) [27]. If a study used multiple eligible measures of functioning or QOL, we extracted data from specific scales using a predefined hierarchy of functioning and QOL measurement scales (Supplementary Table S2). If a study presented data for more than one time point, data for the final time point were analyzed.

### Data extraction and quality assessment

Two independent researchers (TT, BY) extracted the data and assessed the risk of bias. The researchers extracted key study characteristics independently using a standardized data abstraction form, which included diagnostic criteria, drug therapy, treatment duration, age range, mean age, male to female ratio, recruitment location, functioning and QOL measures, number of samples used to measure functioning and QOL, and funding source. We assessed the study risk of bias using the Risk of Bias Tool from the Cochrane Handbook [28]. Studies were classified as having a high risk of bias if two or more domains were rated as having a high risk of bias. Studies were classified as having a low risk of bias if five or more domains were rated as having a low risk of bias, none were rated as having a high risk of bias, and all other cases were considered to have an unknown risk of bias. Disagreements were resolved by a third researcher (XZ).

### Subgroup and sensitivity analyses

To determine whether effectiveness on functioning varied in terms of the moderator variables, we conducted subgroup analyses for medication type (first-generation antidepressants [FGAs; nortriptyline, desipramine, and imipramine] vs. second-generation antidepressants [SGAs; fluoxetine, paroxetine, sertraline, escitalopram, citalopram, and nefazodone]), age (children [aged 6–12 years] vs. adolescents [aged 13–18 years]), sample size (≤100 vs. >100), sex ratio (<1 vs. ≥1), treatment duration (≤8 weeks vs. >8 weeks), and funding source (with vs. without). We used a random-effects model within these subgroups using Review Manager 5.3.5 software (Copenhagen, Denmark). We also conducted sensitivity analyses by excluding trials with treatment duration <8 weeks, trials that allowed concurrent psychotherapy during antidepressant treatment, and trials that used any self-rated scales. The analysis was conducted using Review Manager 5.3.5 with a random-effects model. No subgroup or sensitivity analyses were conducted for QOL because of limited data.

### Statistical analysis

We calculated effect sizes and pooled effect estimates across studies weighted by the inverse variance of each effect size using Review Manager 5.3.5. Standardized mean differences (SMD) with 95% confidence intervals (CIs) were calculated for the effect estimate. A random-effects model was used, and heterogeneity was evaluated using the *I*^2^ statistic. To assess the potential presence of publication bias, funnel plots and Egger’s tests were performed using Review Manager 5.3.5 and Stata 16.0 (College Station, TX, USA), respectively.

## Results

### Study selection and characteristics

**Fig. 1:**
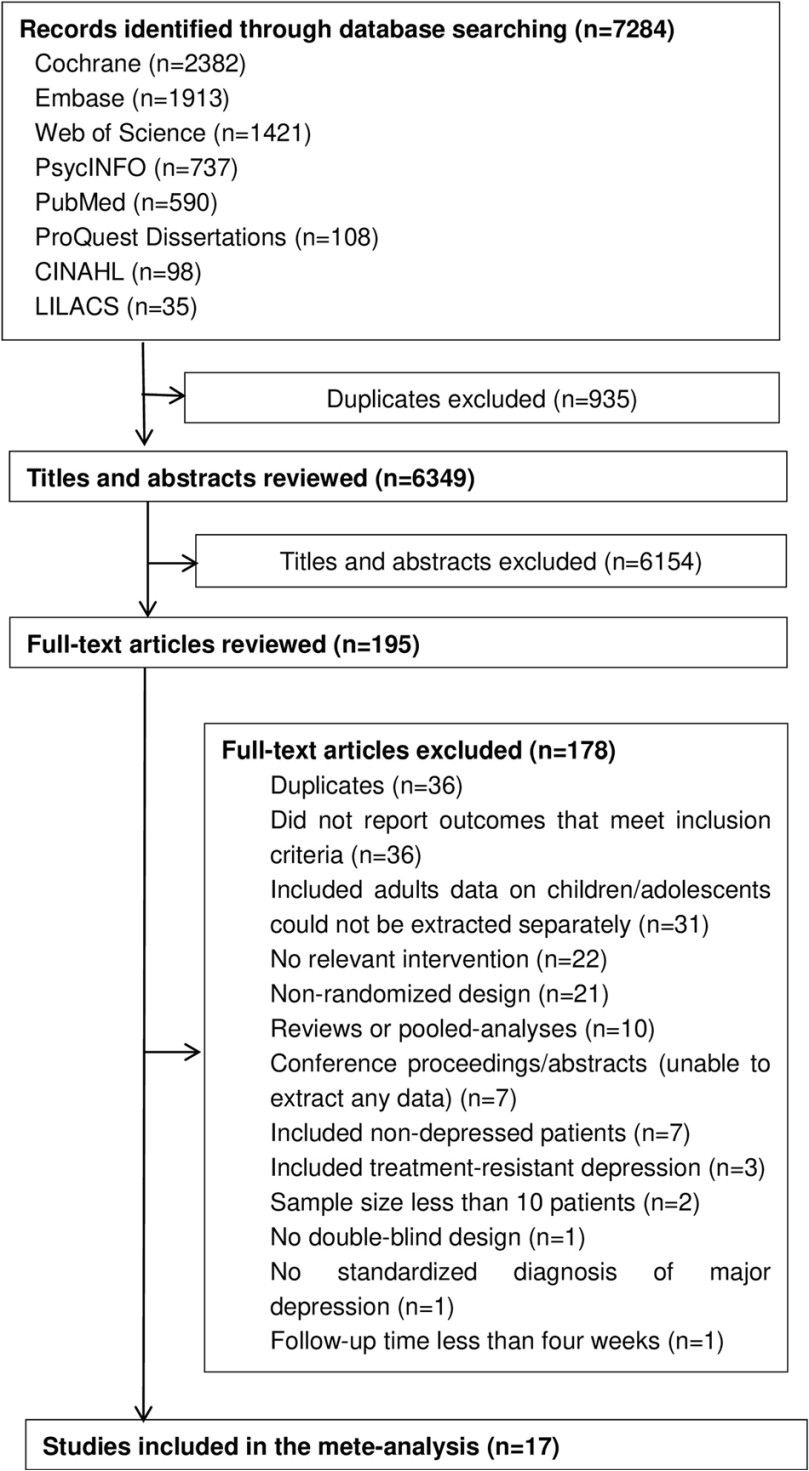
Flowchart of study selection. Of the 7284 relevant studies, 935 were excluded because of duplication, 6154 were excluded because their titles and abstracts did not meet the inclusion criteria, and 178 studies were excluded in full-text screening. 17 RCTs were finally included.

The number of studies excluded at each study stage and the reasons for exclusion are shown in Fig. 1. Of the 7284 relevant studies, 6154 were excluded because their titles and abstracts did not meet the inclusion criteria, and 195 studies were selected for full-text screening. Finally, we included 17 RCTs that compared three FGAs (nortriptyline, desipramine, and imipramine) and six SGAs (fluoxetine, paroxetine, sertraline, escitalopram, citalopram, and nefazodone) with placebo.

The characteristics of the selected studies are shown in Table 1. A total of 2537 participants were included across all trials. In 5 RCTs, 272 participants were studied to compare FGAs with placebo (nortriptyline: two studies with 81 participants; desipramine: one study with 34 participants; and imipramine: two studies with 157 participants). In 13 RCTs, 2327 participants were randomly assigned to SGAs or placebo (fluoxetine: five studies with 526 participants; paroxetine: three studies with 502 participants; sertraline: one study with 364 participants; citalopram: one study with 168 participants; escitalopram: two studies with 572 participants; and nefazodone: one study with 195 participants). The mean age of participants was 13.7 years and ranged from 6 to 18 years. The mean sample size was 169 participants and ranged between 23 and 376 participants. Female participants comprised 53.7% (*n* = 1532) of the sample population. The median treatment duration was 8 weeks and ranged from 5 to 12 weeks. All RCTs measured functioning; 14 studies used the CGAS, two used the GAF, and one used the AFC. Only four RCTs with 926 participants measured QOL. Two studies used the PQ-LES-Q, one used the EuroQol, and one used the SIP.

**Table 1:** Characteristics of included studies.

	Diagnostic criteria	Drug therapy, *n* (drug dose)	Treatment duration (weeks)	Age range (mean age)	Male/female ratio	Recruitment location	Functioning measures	Number of samples measuring functioning	QOL measures	Number of samples measuring QOL	Funding source
Almeida-Montes (2005)	DSM-IV-TR	Fluoxetine, 12 (20 mg/d); Pill-PBO, 11	6	8–14 (11.5)	1.86	Mexico	CGAS (clinician rated)	16 (Fluoxetine, 7; Pill-PBO, 9)	No	No	No
Emslie (1997)	DSM-III-R	Fluoxetine, 48 (20 mg/d); Pill-PBO,48	8	7–17 (12.4)	1.18	USA	CGAS (clinician-rated)	96 (Fluoxetine, 48; Pill-PBO,48)	No	No	No
Emslie (2002a)	DSM-IV	Fluoxetine, 109 (10–20 mg/d); Pill-PBO, 110	9	8–18 (12.7)	1.03	USA	GAF (clinician-rated)	190 (Fluoxetine, 104; Pill-PBO, 86)	No	No	EliLilly
Findling (2009)	DSM-IV	Fluoxetine, 18 (10–20 mg/d); Pill-PBO, 16	8	12–17 (16.5)	5.80	USA	CGAS (clinician-rated)	30 (Fluoxetine, 16; Pill-PBO, 14)	No	No	Eli Lilly
March (2004)	DSM-IV	Fluoxetine, 109 (10–40 mg/d); Pill-PBO, 112	12	12–17 (14.6)	0.84	USA	CGAS (clinician-rated)	194 (Fluoxetine, 98; Pill-PBO, 96)	PQ-LES-Q	190	No
Berard (2006)	DSM-IV	Paroxetine, 187 (20–40 mg/d); Pill-PBO, 99	12	13–18 (15.6)	0.50	Belgium, Italy, Spain, United Kingdom, Holland, Canada, South Africa, United Arab Emirates, Argentina and Mexico	CGAS (clinician-rated)	193 (Paroxetine, 127; Pill-PBO, 66)	EUROQOL	184	GlaxoSmithKline
Emslie (2006)	DSM-IV	Paroxetine, 104 (10–50 mg/d); Pill-PBO, 102	8	7–17 (12.0)	1.14	USA and Canada	GAF (clinician-rated)	187 (Paroxetine, 92; Pill-PBO, 95)	No	No	GlaxoSmithKline
Noury (2015)	DSM-III-R	Paroxetine, 93 (20–60 mg/d); Imipramine, 95 (200–300 mg/d); Pill-PBO, 87	8	12–18 (14.9)	0.61	USA and Canada	AFC (self-rated)	179 (Paroxetine, 60; Imipramine, 57; Pill-PBO, 62)	SIP	188	GlaxoSmithKline
Wagner (2003)	DSM-IV	Sertraline, 189 (50–200 mg/d); Pill-PBO, 187	10	6–17 (NA)	0.96	USA, India, Canada, Costa Rica and Mexico	CGAS* (self-rated)	364 (Sertralin, 185; Pill-PBO, 179)	PQ-LES-Q	364	Pfizer
Wagner (2004)	DSM-IV	Citalopram, 93 (20–40 mg/d); Pill-PBO, 85	8	7–17 (12.1)	0.87	USA	CGAS (clinician-rated)	168 (Citalopram,87; Pill-PBO, 81)	No	No	Forest Laboratories
Emslie (2009)	DSM-IV	Escitalopram,158 (10–20 mg/d); Pill-PBO, 158	8	12–17 (14.6)	0.70	USA	CGAS (clinician-rated)	311 (Escitalopram, 154; Pill-PBO, 157)	No	No	Forest Laboratories
Wagner (2006)	DSM-IV	Escitalopram,132 (10–20 mg/d); Pill-PBO, 136	8	6–17 (12.3)	0.93	USA	CGAS (clinician-rated)	261 (Escitalopram, 129; Pill-PBO, 132)	No	No	Forest Laboratories
Emslie (2002b)	DSM-IV	Nefazodone, 99 (100–400 mg/d); Pill-PBO, 96	8	12–17 (NA)	0.70	NA	CGAS (clinician-rated)	195 (Nefazodone,99; Pill-PBO, 96)	No	No	Bristol-Myers Squibb
Geller (1990)	DSM-III and RDC	Nortriptyline, 12 (10–140 mg/d); Pill-PBO, 19	8	12–17 (14.3)	1.21	USA	CGAS (clinician-rated)	31 (Nortriptyline, 12; Pill-PBO, 19)	No	No	No
Geller (1992)	DSM-III and RDC	Nortriptyline, 30 (10–140 mg/d); Pill-PBO, 30	8	6–12 (9.7)	2.33	NA	CGAS (clinician-rated)	50 (Nortriptyline, 26; Pill-PBO, 24)	No	No	No
Klein (1998)	DSM-III-R	Desipramine, 23 (50–300 mg/d); Pill-PBO, 22	6	13–18 (15.7)	0.50	USA	CGAS (clinician-rated)	34 (Desipramine, 17; Pill-PBO, 17)	No	No	No
Puig-Antich (1987)	K-SADS and RDC	Imipramine, 20 (3.25–5 mg/d per kg); Pill-PBO, 22	5	6–12 (9.1)	1.53	USA	CGAS (clinician-rated)	38 (Imipramine, 16; Pill-PBO, 22)	No	No	No

*Note*: Definitions of abbreviations are listed below:

PBO, placebo; NA, not available; QOL, quality of life.

*Functioning measures*: CGAS, Children’s Global Assessment Scale; GAF, global assessment function; AFC, autonomous functioning checklist. *The author stated in the published article that they used CGAS as a self-rated scale.

*QOL measures*: PQ-LES-Q, Pediatric Quality of Life Enjoyment and Satisfaction Questionnaire; EuroQol, European Quality of Life Scale; SIP, sickness impact profile.

*Depression measures*: DSM, Diagnostic and Statistical Manual of Mental Disorders; DSM-III-R, Diagnostic and Statistical Manual of Mental Disorders, Third Edition, Revised; DSM-IV-TR, Diagnostic and Statistical Manual of Mental Disorders, Fourth Edition, Text Revision; K-SADS, Kiddie-Schedule for Affective Disorders and Schizophrenia for School-Age Children; RDC, Research Diagnostic Criteria.

### Quality assessment

The risk of bias assessment showed that 2 studies (11.8%) had a low risk of bias and 12 studies (70.6%) had an unknown risk. In addition, 3 studies (17.6%) had high reporting bias owing to the presence of reporting bias, attrition bias, and other bias. Supplementary Fig. S1 shows the detailed assessment of risk of bias. We used a funnel plot (Supplementary Fig. S2) and Egger’s tests to detect publication bias (*p* = 0.530), which indicated that there was no significant publication bias in any of the included studies.

### Treatment effects on functioning and QOL

**Fig. 2:**
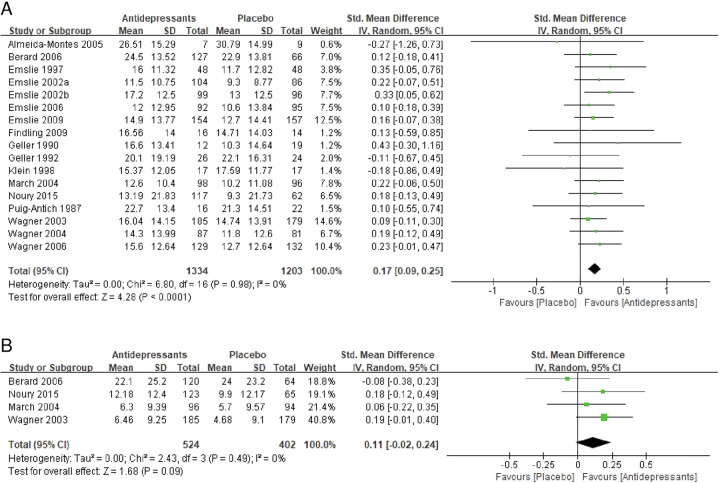
Meta-analysis of the primary functioning and QOL outcomes. **A** Forest plot of the standardized mean difference (SMD) of the change in functioning scale scores for the comparison between antidepressants and placebo. **B** Forest plot of the SMD for the change in quality-of-life scale scores for the comparison between antidepressants and placebo. SD standard deviation, CI confidence interval.

Compared with the placebo group, the antidepressant group showed significant positive effects for functioning (SMD = 0.17, 95% CI = 0.09–0.25, *p* < 0.0001, Fig. 2A), with no significant heterogeneity (*I*^2^ = 0, *p* = 0.977, Fig. 2A). Regarding QOL, compared with the placebo group, the antidepressant group showed no significant effects (SMD = 0.11, 95% CI = −0.02 to 0.24, *p* = 0.093, Fig. 2B) and no significant heterogeneity (*I*^2^ = 0, *p* = 0.488, Fig. 2B).

### Subgroup analyses

Subgroup analyses were only conducted for the functioning outcome because of limited QOL data. The results of the subgroup analyses for functioning are shown in Table 2. Statistically significant positive effects on improvements in functioning were observed for SGAs (SMD = 0.18, 95% CI = 0.10–0.27, *p* < 0.001), adolescents (SMD = 0.19, 95% CI = 0.07–0.31, *p* = 0.001), trials with a sample size >100 (SMD = 0.18, 95% CI = 0.09–0.26, *p* < 0.001), and trials with funding (SMD = 0.17, 95% CI = 0.09–0.26, *p* < 0.001), but not for FGAs, children, trials with a sample size ≤100, and trials without funding. No significant differences were found in the subgroup analyses of sex ratio or treatment duration. Regarding specific classes of antidepressants, fluoxetine (SMD = 0.23, 95% CI = 0.05–0.40, *p* = 0.010), escitalopram (SMD = 0.19, 95% CI = 0.02–0.35, *p* = 0.024), and nefazodone (SMD = 0.33, 95% CI = 0.05–0.62, *p* = 0.020) were significantly more effective for functioning, whereas the other antidepressants showed no significant effect on functioning.

**Table 2:** Subgroup analyses for the primary outcome of functioning.

	Trials number subgroups (sample size)	Overall effect		Heterogeneity	
		SMD (95% CI)	*P*	*I*^*2*^ (%)	*P*
*Medication type*					
First-generation antidepressants	5 (272)	0.06 [−0.18, 0.30]	0.610	0	0.750
Nortriptyline	2 (81)	0.09 [−0.35, 0.53]	0.697	25	0.247
Desipramine	1 (34)	−0.18 [−0.86, 0.49]	0.596	–	–
Imipramine	2 (157)	0.10 [−0.21, 0.42]	0.522	0	0.991
Second-generation antidepressants	13 (2327)	0.18 [0.10, 0.27]	<0.001	0	0.978
Fluoxetine	5 (526)	0.23 [0.05, 0.40]	0.010	0	0.844
Paroxetine	3 (502)	0.14 [−0.03, 0.32]	0.112	0	0.807
Sertraline	1 (364)	0.09 [−0.11, 0.30]	0.379	–	–
Escitalopram	2 (572)	0.19 [0.02, 0.35]	0.024	0	0.663
Nefazodone	1 (195)	0.33 [0.05, 0.62]	0.020	–	–
Citalopram	1 (168)	0.19 [−0.12, 0.49]	0.226	–	–
*Age*					
Children	2 (88)	−0.02 [−0.44, 0.40]	0.920	0	0.632
Adolescents	8 (1167)	0.19 [0.07, 0.31]	0.001	0	0.884
*Sample size*					
≤100 participants	7 (295)	0.13 [−0.10, 0.36]	0.271	0	0.677
>100 participants	10 (2242)	0.18 [0.09, 0.26]	<0.001	0	0.977
*Male/Female ratio*					
≥1	8 (638)	0.17 [0.01, 0.32]	0.038	0	0.847
<1	9 (1899)	0.17 [0.08, 0.26]	<0.001	0	0.907
*Treatment duration*					
≤8 weeks	13 (1596)	0.18 [0.09, 0.28]	<0.001	0	0.926
>8 weeks	4 (941)	0.15 [0.02, 0.28]	0.023	0	0.840
*Funding source*					
With	10(2078)	0.17 [0.09, 0.26]	<0.001	0	0.979
Without	7(459)	0.17 [−0.02, 0.35]	0.072	0	0.644

*SMD* standardized mean difference, *CI* confidence interval.

### Sensitivity analyses

**Fig. 3:**
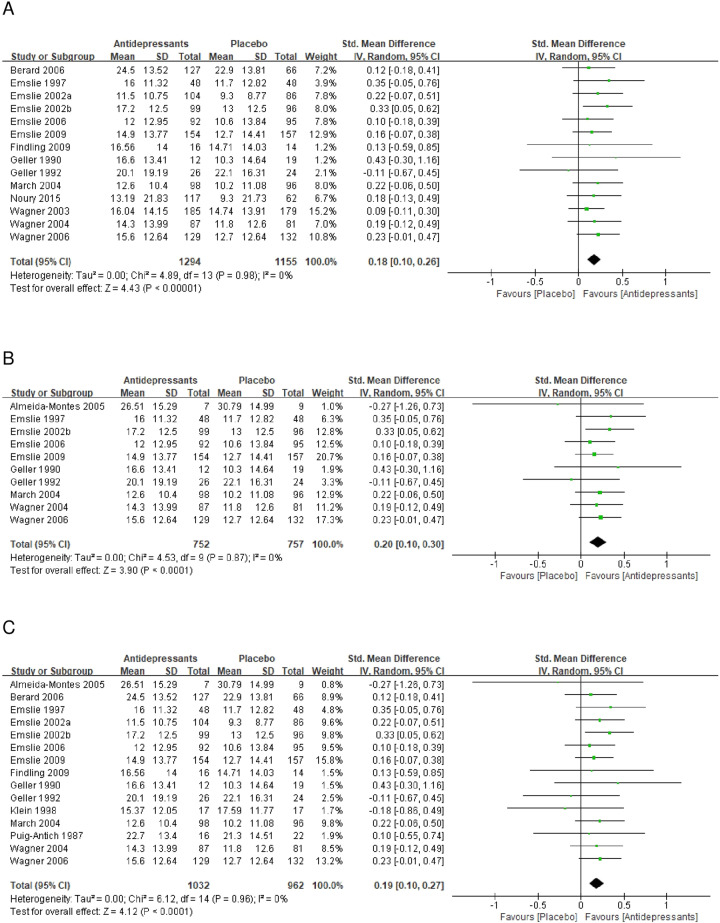
Meta-analysis of the sensitivity analysis. **A** Forest plot of the standardized mean difference (SMD) of the change in functioning scale scores for the comparison between antidepressants and placebo when excluding studies with treatment duration <8 weeks. **B** Forest plot of the SMD of the change in functioning scale scores for the comparison between antidepressants and placebo when excluding studies allowing concurrent psychotherapy during treatment. **C** Forest plot of the SMD of the change in functioning scale scores for the comparison between antidepressants and placebo when excluding studies using self-rated scales. *SD* standard deviation, *CI* confidence interval.

Sensitivity analyses were only conducted for the functioning outcome because of limited QOL data. There was a minimal change to the effect size with no significant heterogeneity after excluding trials with treatment duration <8 weeks (SMD = 0.18, 95% CI = 0.10–0.26, *p* < 0.0001, Fig. 3A), trials that allowed concurrent psychotherapy during treatment (SMD = 0.20, 95% CI = 0.10–0.30, *p* < 0.0001, Fig. 3B), and trials that used self-rated scales (SMD = 0.19, 95% CI = 0.10–0.27, *p* < 0.0001, Fig. 3C).

## Discussion

To the best of our knowledge, this is the first meta-analysis of RCTs investigating antidepressants for functioning and QOL in children and adolescents with MDD. This study demonstrated that antidepressants have positive effects on functioning in children and adolescents with MDD, but found no positive outcomes for QOL. Subgroup analyses showed that SGAs, especially fluoxetine, escitalopram, and nefazodone, significantly improved functioning, whereas FGAs did not.

Our results for functioning are consistent with those of previous studies on functioning outcomes of antidepressants in adults with MDD [29]. A meta-analysis of studies in adults with MDD reported a similar effect on functioning (Hedges’ *g* = 0.31, 95% CI = 0.26–0.36) [29]. However, this previous meta-analysis focused on adults investigated not only the antidepressants included in the present study but also many other drugs not frequently used with children and adolescents with MDD, such as levomilnacipran, desvenlafaxine, and amitriptyline. Regarding QOL, a meta-analysis of studies of adults with MDD reported that pharmacotherapy significantly improved QOL (Hedges’ *g* = 0.79, 95% CI = 0.67–0.91, *p* < 0.01) [30], which differs from our own findings. There are several possible reasons for this difference. First, although symptom severity did not affect the recovery of QOL, it did account for a proportion of the variance in QOL scores [31]. To improve QOL in pediatric MDD, it may be necessary to combine antidepressants with cognitive–behavioral therapy, a treatment that showed effectiveness in the Treatment for Adolescents with Depression Study (TADS) [5]. Second, longer treatment duration (median: 10 weeks) in the adult meta-analysis may have contributed to a more effective outcome compared with our results. Third, only a small number of RCTs included in the present study investigated QOL, which probably reduced the statistical validity [32]. Moreover, recent studies indicate that narrative medicine approaches can help to increase patient treatment adherence and improve QOL in some diseases, by constructing positive physician–patient relationships based on listening to and understanding the experiences patients narrate [33, 34]. Future studies could use narrative medicine to develop QOL-targeted treatments, which may introduce new ideas for improving QOL in children and adolescents with MDD.

The subgroup analyses showed that SGAs (especially fluoxetine, escitalopram, and nefazodone) were associated with a significant improvement in functioning, whereas FGAs were not. However, this difference was in contrast to a meta-analysis of adult studies [29] that showed that the effects of TCAs (FGAs) and SSRIs (SGAs) on adult functioning were comparable. Several factors may explain the differing effects between juveniles and adults. First, TCAs are effective for treating adult depressive symptoms [35] but ineffective for treating depressive symptoms in children and adolescents [36]. Second, FGAs induce more adverse effects than do SGAs, which may negatively affect the functioning of children and adolescents [37, 38]. Of the nine antidepressants included in our study, only fluoxetine, escitalopram, and nefazodone significantly improved functioning, and all three are SGAs. The outcomes of these three antidepressants observed here are consistent with their positive effects on depressive symptoms in pediatric populations [39–41]. However, the effect of nefazodone on functioning should be interpreted with caution because our sample size was relatively small. Moreover, antidepressants significantly improved functioning in the adolescent subgroup but not in the child subgroup. This difference between children and adolescents could be explained by previous findings indicating that many antidepressants have a greater effect on depressive symptoms in adolescents than in children [42, 43]. However, interpretation of the results for the child subgroup requires caution because the sample size was small. Different outcomes were also found between studies with and without funding sources. This may reflect conflicts of interest in some studies, which could lead to more favorable efficacy results and conclusions that reflect the sponsors’ interests [44]. Additionally, trials with funding may have larger sample sizes.

This study had several limitations. First, for some of the analyses, the number of studies and sample sizes may be insufficient to permit the generalizability of results, especially for the QOL analysis, which only included four studies. Second, most included studies had an unknown risk of bias. Third, there were no follow-up data; therefore, we could not examine the long-term effects of antidepressants on both functioning and QOL. This would require longitudinal study designs because patients may experience functional impairments for many months after achieving remission from depression [45]. Fourth, the treatment duration in some included studies may have been too short to observe the functioning outcome. More trials with longer treatment durations for children and adolescents with MDD may be needed to confirm this finding. Fifth, most included functioning scales were clinician-report scales, which may not generate patient-centered and youth-guided diagnosis and treatment in clinical practice [6]. However, the sensitivity analysis excluding studies using self-report scales showed no significant difference. Sixth, seven included RCTs allowed concurrent psychological treatment during antidepressant treatment, such as family therapy and supportive psychotherapy. This may have supplemented the antidepressant effects. However, a sensitivity analysis excluding these studies showed no significant difference. Seventh, based on the evidence from a recent study in adults [46], the effects of antidepressants on functioning and QOL in children and adolescents with MDD might be also overestimated due to potential observers’ bias of functioning and QOL outcomes in our included trials, which would be an interesting topic in further study. Finally, to reduce heterogeneity across studies, we excluded patients with treatment-resistant depression, which may have led to an overestimation of effects because of the omission of a difficult-to-treat population.

In conclusion, this meta-analysis provides evidence that currently used antidepressants, especially SGAs (fluoxetine and escitalopram), are efficacious for improving functioning in children and adolescents with MDD, but are ineffective in improving QOL. Our findings offer valuable and comprehensive evidence for pharmacotherapy decisions in clinical practice to improve functioning in children and adolescents with MDD. However, more well-designed clinical studies using large samples are needed.

## Supplementary information

Supplemental Table S1

Supplemental Table S2

supplementary figure legends

Supplemental Figure S1

Supplemental Figure S2
