# Supplementary material for: Effect of antidepressants on functioning and quality of life outcomes in children and adolescents with major depressive disorder: a systematic review and meta-analysis
Source: Transl Psychiatry. 2022 May 4;12:183. doi: 10.1038/s41398-022-01951-9 (PMC9068747; doi:10.1038/s41398-022-01951-9)
Supplement: Supplementary file 1 — Supplemental Table S1 [file 41398_2022_1951_MOESM1_ESM.docx]

**Table S1. Number of citations by each database***

| **Databases:** |  | **Citations** |
| --- | --- | --- |
| Cochrane |  | 2,382 |
| Embase |  | 1,913 |
| Web of Science |  | 1,421 |
| PsychInfo |  | 737 |
| PubMed |  | 590 |
| Proquest |  | 108 |
| CINAHL |  | 98 |
| LILACS |  | 35 |
| **Total (databases)** |  | **7,284** |

***Explicit search strateg**y: title/abstract = (depress* or dysthymi* or “mood disorder*” or “affective disorder*”) AND (adolesc* or child* or boy* or girl* or juvenil* or minors or paediatri* or pediatri* or pubescen* or school* or student* or teen* or young or youth* or preschool or pre-school) AND (antidepressant* or 'selective serotonin reuptake inhibitor*' or ssri or ssris or fluoxetine or fluvoxamine or paroxetine or sertraline or citalopram or escitalopram or vortioxetine or 'serotonin norepinephrine reuptake inhibitor*' or snri or snris or duloxetine or venlafaxine or desvenlafaxine or milnacipran or levomilnacipran or mianserin or nefazodone or trazodone or vilazodone or bupropion or reboxetine or agomelatine or 'noradrenergic and specific serotonergic antidepressant*' or nassa or nassas or mirtazapine or tca or tcas or tricyclic or amersergide or amineptine or amitriptyline or amoxapine or butriptyline or chlorpoxiten or clomipramine or clorimipramine or demexiptiline or desipramine or dibenzipin or dothiepin or doxepin or imipramine or lofepramine or melitracen or metapramine or nortriptyline or noxiptiline or opipramol or protriptyline or quinupramine or tianeptine or trimipramine)

**MeSH search strategy:** MeSH = (“depressive disorder” or “dysthymic disorder” or “mood disorders” or “affective disorders”) AND (child or adolescent) AND (“antidepressive agents”)
