# Supplementary material for: Effect of antidepressants on functioning and quality of life outcomes in children and adolescents with major depressive disorder: a systematic review and meta-analysis
Source: Transl Psychiatry. 2022 May 4;12:183. doi: 10.1038/s41398-022-01951-9 (PMC9068747; doi:10.1038/s41398-022-01951-9)
Supplement: Supplementary file 2 — Supplemental Table S2 [file 41398_2022_1951_MOESM2_ESM.docx]

Table S2. Hierarchy of functioning and QOL measures

| Hierarchy | Functioning Measure | Abbreviation |
| --- | --- | --- |
| 1 | Children’s Global Assessment Scale | CGAS |
| 2 | Global Assessment Function | GAF |
| 3 | Autonomous Functioning Checklist | AFC |
| 4 | Health of the Nation Outcome Scales for Children and Adolescents | HoNOSCA |
| 5 | Self-Perception Profile | SPP |

| Hierarchy | QOL Measure | Abbreviation |
| --- | --- | --- |
| 1 | Pediatric Quality of Life Enjoyment and Satisfaction Questionnaire | PQ-LES-Q |
| 2 | The World Health Organization Quality of Life Brief Version | WHOQOL-BREF |
| 3 | EuroQol five-dimension scale | EQ-5D |
| 4 | The MOS 36-item short-form health survey | SF-36 |
| 5 | Nottingham health profile | NHP |
| 6 | Sickness Impact Scale | SIP |

Where different functioning or QOL measures were used for the purpose of pooling results, we chose the single best available measure according to a hierarchy based on our operational definition of functioning or QOL.
