# Supplementary material for: Effect of antidepressants on functioning and quality of life outcomes in children and adolescents with major depressive disorder: a systematic review and meta-analysis
Source: Transl Psychiatry. 2022 May 4;12:183. doi: 10.1038/s41398-022-01951-9 (PMC9068747; doi:10.1038/s41398-022-01951-9)
Supplement: Supplementary file 3 — supplementary figure legends [file 41398_2022_1951_MOESM3_ESM.docx]

**Figure S1. Risk of bias assessment**

+ = low risk of bias; ? = unknown risk of bias; − = high risk of bias. Risk of bias owing to blinding of outcome assessment refers to the functioning and quality of life measures.

**Figure S2. Funnel plot**

SE = standard error; SMD = standard mean difference.
