# Supplementary material for: Effect of antidepressants on functioning and quality of life outcomes in children and adolescents with major depressive disorder: a systematic review and meta-analysis
Source: Transl Psychiatry. 2022 May 4;12:183. doi: 10.1038/s41398-022-01951-9 (PMC9068747; doi:10.1038/s41398-022-01951-9)
Supplement: Supplementary file 4 — Supplemental Figure S1 [file 41398_2022_1951_MOESM4_ESM.pdf]

	Random sequence generation (selection bias)	Allocation concealment (selection bias)	Blinding of participants and personnel (performance bias)	Blinding of outcome assessment (detection bias)	Incomplete outcome data (attrition bias)	Selective reporting (reporting bias)	Other bias	Total bias
Almeida-Montes 2005	+	+	?	+	−	?	?	?
Berard 2006	+	+	+	?	?	+	?	?
Emslie 1997	+	?	+	+	+	−	?	?
Emslie 2002a	+	?	+	?	?	−	−	−
Emslie 2002b	?	?	?	?	?	?	?	?
Emslie 2006	+	?	?	?	+	+	?	?
Emslie 2009	?	?	?	?	+	−	+	?
Findling 2009	+	+	?	+	+	?	?	?
Geller 1990	?	?	+	−	?	?	?	?
Geller 1992	?	?	+	+	+	?	?	?
Klein 1998	?	?	?	?	?	?	?	?
March 2004	+	+	+	+	+	−	?	+
Noury 2015	+	+	+	+	+	+	+	+
Puig-Antich 1987	+	?	+	+	?	?	?	?
Wagner 2003	+	?	+	?	+	−	−	−
Wagner 2004	?	?	?	?	−	−	?	−
Wagner 2006	?	?	?	?	?	?	+	?
