# Supplementary figures and images for: Effect of antidepressants on functioning and quality of life outcomes in children and adolescents with major depressive disorder: a systematic review and meta-analysis
Source: Transl Psychiatry. 2022 May 4;12:183. doi: 10.1038/s41398-022-01951-9 (PMC9068747; doi:10.1038/s41398-022-01951-9)

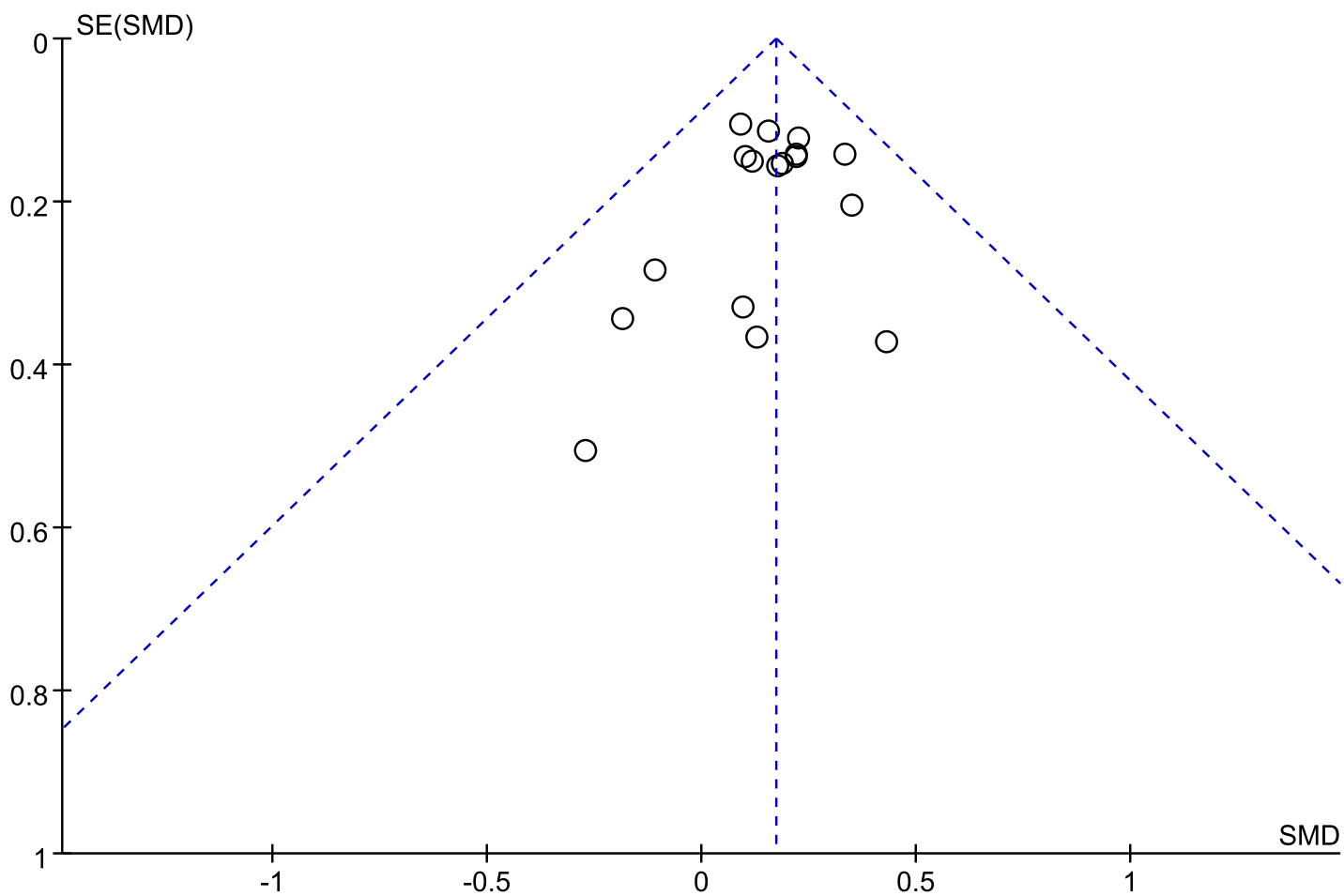

Supplement: Supplementary file 5 — Supplemental Figure S2 [file 41398_2022_1951_MOESM5_ESM.pdf]
